# The neuroanatomical hallmarks of chronic tinnitus in comorbidity with pure-tone hearing loss

**DOI:** 10.1007/s00429-023-02669-0

**Published:** 2023-06-22

**Authors:** Stefan Elmer, Raffael Schmitt, Nathalie Giroud, Martin Meyer

**Affiliations:** 1 Department of Computational Linguistics, Computational Neuroscience of Speech & Hearing, University of Zurich, Zurich, Switzerland; 2 Department of Comparative Language Science, University of Zurich, Zurich, Switzerland; 3 Center for Neuroscience Zurich, University and ETH of Zurich, Zurich, Switzerland; 4 Center for the Interdisciplinary Study of Language Evolution (ISLE), University of Zurich, Zurich, Switzerland; 5 Cognitive Psychology Unit, Alpen-Adria University, Klagenfurt, Austria; 6 Competence Center Language & Medicine, University of Zurich, Zurich, Switzerland

**Keywords:** Tinnitus, Distress, Syndrome, Supra-threshold hearing, Hearing loss, Speech-in-noise, Structural MRI, Cortical volume, Cortical thickness, Cortical surface area

## Abstract

Tinnitus is one of the main hearing impairments often associated with pure-tone hearing loss, and typically manifested in the perception of phantom sounds. Nevertheless, tinnitus has traditionally been studied in isolation without necessarily considering auditory ghosting and hearing loss as part of the same syndrome. Hence, in the present neuroanatomical study, we attempted to pave the way toward a better understanding of the tinnitus syndrome, and compared two groups of almost perfectly matched individuals with (TIHL) and without (NTHL) pure-tone tinnitus, but both characterized by pure-tone hearing loss. The two groups were homogenized in terms of sample size, age, gender, handedness, education, and hearing loss. Furthermore, since the assessment of pure-tone hearing thresholds alone is not sufficient to describe the full spectrum of hearing abilities, the two groups were also harmonized for supra-threshold hearing estimates which were collected using temporal compression, frequency selectivity und speech-in-noise tasks. Regions-of-interest (ROI) analyses based on key brain structures identified in previous neuroimaging studies showed that the TIHL group exhibited increased cortical volume (CV) and surface area (CSA) of the right supramarginal gyrus and posterior planum temporale (PT) as well as CSA of the left middle-anterior part of the superior temporal sulcus (STS). The TIHL group also demonstrated larger volumes of the left amygdala and of the left head and body of the hippocampus. Notably, vertex-wise multiple linear regression analyses additionally brought to light that CSA of a specific cluster, which was located in the left middle-anterior part of the STS and overlapped with the one found to be significant in the between-group analyses, was positively associated with tinnitus distress level. Furthermore, distress also positively correlated with CSA of gray matter vertices in the right dorsal prefrontal cortex and the right posterior STS, whereas tinnitus duration was positively associated with CSA and CV of the right angular gyrus (AG) and posterior part of the STS. These results provide new insights into the critical gray matter architecture of the tinnitus syndrome matrix responsible for the emergence, maintenance and distress of auditory phantom sensations.

## Introduction

Tinnitus is commonly experienced as ringing, hissing or noise in the ears in the absence of any corresponding physical sound source in the environment (Baguley et al. 2013; Cederroth et al. 2019). Such phantom auditory sensations are one of the main otological symptoms frequently associated with pure-tone hearing loss (Axelsson and Ringdahl 1989; Savastano 2008), have an estimated lifetime prevalence in the range of 9–26% (McCormack et al. 2014; Sahlsten et al. 2018), and are supposed to increase in parallel with age-related hearing impairments (Oosterloo et al. 2021; Rosing et al. 2016)**.** Tinnitus is usually characterized by a chronic (> 12 month) course, can be extremely disturbing and bothersome, and is, therefore, often accompanied by psychological distress (Adjamian et al. 2014; Axelsson and Ringdahl 1989; Savastano 2008). Nevertheless, there is reason to believe that the latter discomfort tends to improve over time without mandatory changes in phantom sound characteristics (Vielsmeier et al. 2020; Wallhausser-Franke et al. 2017).

Although the etiology of tinnitus is multifactorial in nature and its pathophysiology remains somewhat elusive (Adjamian et al. 2014), the frequent co-occurrence (~ 90%) of tinnitus and pure-tone hearing loss (Davis and El Rafaie 2000) has led to the assumption that both disorders are anchored to a common origin in the inner ear (Gu et al. 2010). According to this perspective, damage to inner hair cells of the cochlea is supposed to affect neural impulse transmission along the peripheral auditory pathway, and to promote thalamocortical dysrhythmia (Llinas et al. 1999; Meyer et al. 2014). More specifically, as a consequence of deafferentiation following hair cell loss in the cochlea, the auditory thalamus starts generating spontaneous low-frequency oscillations which result in hyperactivity of auditory cortical fields (Llinas et al. 1999). A complementary neural mechanism which has been proposed to contribute to the emergence and maintenance of tinnitus is a dysbalance between excitation and inhibition in the auditory cortex, possibly due to bottom-up input deprivation (Eggermont 2007, 2008; Okamoto et al. 2010). Thereby, deafferentiated neurons in the auditory cortex become sensitive to adjacent frequencies, lose their lateral inhibition properties, and the resulting maladaptive reorganization of tonotopic maps leads to inherent rhythmic oscillations which are manifested in an over-representation of the tinnitus frequency (Eggermont 2007, 2008).

The view that tinnitus is primarily determined by cochlear dysfunctions (Schmidt et al. 2018) has been challenged by single-case reports showing that a dissection of the auditory nerve was not necessarily accompanied by a relief of the symptoms (House and Brackmann 1981). In addition, the absence of measurable pure-tone hearing loss in some individuals suffering from tinnitus raises further questions about the generalizability of the neural principles essentially involved in auditory phantom sensations (Weisz et al. 2006). Hence, tinnitus cannot be attributed exclusively to a dysfunction of the inner ear or to maladaptive plasticity in the auditory cortex, but should rather be considered as a syndrome involving large-scale neural networks and multiple structures of the central nervous system (De Ridder et al. 2011). The plausibility of this argument is strengthened by functional neuroimaging studies showing that tinnitus is associated with aberrant brain activity patterns in cortical–subcortical circuits including both auditory and non-auditory brain regions (Kleinjung and Langguth 2020). While some studies provided evidence for tinnitus-related functional peculiarities in the inferior colliculi, the thalamus, and the auditory cortex (Arnold et al. 1996; Eichhammer et al. 2003; Giraud et al. 1999; Langguth et al. 2006; Lanting et al. 2008; Melcher et al. 2000; Smits et al. 2007), other reported dysfunctional brain activity in limbic regions, the inferior parietal lobe, the anterior cingulate cortex as well as in different subdivision of the prefrontal cortex (Araneda et al. 2018; Carpenter-Thompson et al. 2015; Cheng et al. 2020; Golm et al. 2013; Lanting et al. 2009; Seydell-Greenwald et al. 2012). Drawing on this background, it is conceivable that pathophysiological mechanisms in peripheral auditory structures constitute a critical step in the development of tinnitus, while the consequent altered signal transmission to the central auditory system as well as maladaptive plastic changes in several cortical brain areas are responsible for its manifestation and maintenance (Lenarz et al. 1993).

Since brain functions are determined, among other factors, by the underlying neural architecture (Batista-Garcia-Ramo and Fernandez-Verdecia 2018; Lehericy et al. 2000), in the last decade, more and more studies began to examine tinnitus-related gray matter changes using voxel-based (VBM) or surface-based morphometry (SBM) (Allan et al. 2016; Liu et al. 2018; Meyer et al. 2016; Schecklmann et al. 2013; Scott-Wittenborn et al. 2017; Wei et al. 2021; Muhlau et al. 2006), whereas the microstructural properties of white matter pathways have commonly been estimated by means of diffusion tensor imaging (DTI) techniques (Benson et al. 2014; Husain et al. 2011; Schmidt et al. 2018). Currently, two main avenues of research have been pursued to characterize the neuroanatomical hallmarks of tinnitus. A first approach relies on the comparison between individuals with and without tinnitus who demonstrate pure-tone audiometric profiles in the normative range (pure-tone thresholds < 25 dB hearing loss) (Aldhafeeri et al. 2012; Besteher et al. 2019; Landgrebe et al. 2009; Muhlau et al. 2006; Schmidt et al. 2018). Although such a procedure is particularly fruitful to control for the influence of hearing acuity, it does not take into account the high comorbidity of tinnitus and pure-tone hearing loss (Davis and El Rafaie 2000). Therefore, a second alternative strategy consists of considering both auditory-related disorders as a single entity rather than as separate or confounding parts (Allan et al. 2016; Benson et al. 2014; Schneider et al. 2009; Vanneste et al. 2015). In this sense, the nexus between tinnitus and pure-tone hearing loss can be addressed, for example, by comparing two groups of individuals with and without phantom manifestations but both exhibiting pure-tone hearing loss (Allan et al. 2016; Benson et al. 2014; Vanneste et al. 2015).

The first experimental approach aiming at mapping tinnitus-related gray matter characteristics in normal-hearing individuals was used, for example, by Mühlau and colleagues (Muhlau et al. 2006) who performed whole-brain VBM analyses and revealed reduced gray matter volume in the subcallosal area of participants affected by tinnitus. Additional regions-of-interest (ROI) analyses only including auditory brain regions also brought to light tinnitus-related increased gray matter density in the right posterior thalamus and the medial geniculate nucleus. However, using a comparable morphometric approach, Landgrebe and colleagues (Landgrebe et al. 2009) were unable to replicate the results, and instead the authors found reduced gray matter volume in individuals with tinnitus in the right inferior colliculus and in the left hippocampus. In a further VBM study, Besteher and colleagues (Besteher et al. 2019) used a ROI-based approach and found decreased gray matter volume as a function of tinnitus in the parahippocampal cortex. Noticeably, with the advent of new computational neuroanatomy tools, some studies harked back to the SBM technique, which bears the advantage of estimating multiple gray matter indices that can be considered as independent traits with different levels of heritability (Rakic 1995, 2000), namely cortical thickness (CT), cortical surface area (CSA) and cortical volume (CV) (Goto et al. 2022). Taking advantage of this approach, Aldhafeeri and colleagues (Aldhafeeri et al. 2012) performed whole-brain and ROI-based SBM analyses, and reported reduced CT in the tinnitus group in a diffuse neural network including the superior, middle and inferior frontal gyri, the anterior cingulate cortex, the cingulate gyrus, the superior, middle and inferior temporal gyri as well as the right primary auditory cortex. Although the results of all these previous anatomical studies are highly heterogeneous, they indicate that tinnitus should be considered as a multidimensional phenomenon related to gray matter peculiarities in sensory, cognitive as well as limbic areas.

The second main branch of research introduced above addressed tinnitus and pure-tone hearing loss as parts of a syndrome, and focused on the comparison between hearing-impaired participants with and without tinnitus (Allan et al. 2016; Benson et al. 2014; Boyen et al. 2013; Husain et al. 2011; Leaver et al. 2012; Schmidt et al. 2018). For example, Boyen and colleagues (Boyen et al. 2013) combined whole-brain and ROI-based VBM analyses, and found increased CV in the left primary auditory cortex of participants with tinnitus compared to the control group. However, this result was only manifested in the ROI analysis, leading to suggest that the whole-brain approach was not sensitive enough or too conservative to detect small between-group differences. In contrast, the whole-brain VBM analysis of Husain and colleagues (Husain et al. 2011) uncovered increased CV in the bilateral superior and medial frontal gyri of participants affected by tinnitus and hearing loss compared to control participants only characterized by hearing loss. In a further structural magnetic resonance imaging (MRI) study which included both VBM and SBM analyses, Leaver and colleagues (Leaver et al. 2012) found that the chronic tinnitus group exhibited reduced CSA in the ventromedial prefrontal cortex, even though this effect was not related to tinnitus distress, depression, anxiety, or noise sensitivity. However, tinnitus distress positively correlated with CT in the anterior insula, while anxiety and depression were negatively associated with CT in the subcallosal area, irrespective of group affiliation. In addition, Allan and co-workers (Allan et al. 2016) applied whole-brain VBM and SBM analyses, and the results indicated decreased CT in the left auditory cortex of tinnitus-affected participants, whereas the VBM analyses did not reveal significant CV differences between the two groups.

Several reasons may account for the somewhat contradictory results emerging from neuroanatomical studies on tinnitus which used different morphometric approaches to identify the crucial gray matter ‘nodes’ responsible for auditory phantom sensations and tinnitus-related distress. A first consideration is that previous work partially included groups that were poorly matched on multiple dimensions, including sample size, age, sex, handedness, education as well as hearing loss. Furthermore, the majority of studies focusing on the joint manifestation of tinnitus and hearing loss only used a pure-tone threshold screener to document the integrity of extra-cortical sources instead of providing a global hearing assessment that takes into account the critical influence of supra-threshold hearing estimates (Fullgrabe et al. 2015; Giroud et al. 2018). Such a drawback is somewhat surprising, than the view that the exclusive assessment of pure-tone thresholds is not enough to describe the full spectrum of hearing capabilities in older age is not at all novel (Dubno et al. 2002; Fullgrabe et al. 2015; Giroud et al. 2018; Humes 2019). Accordingly, proper hearing should rather be considered as an active and integrative process that enables adaptive listening behavior, and that relies on the interplay between the auditory periphery, the central auditory system and brain regions involved in the regulation of higher cognitive functions (Fulton et al. 2015; Giroud et al. 2018). In contrast to pure-tone hearing, supra-threshold hearing more specifically refers to the spectral and temporal resolution properties of the central auditory system that can be measured, for example, by means of frequency selectivity (FS) and temporal compression (TC) tasks (Giroud et al. 2018; Lecluyse et al. 2013). Furthermore, tasks such as speech-in-noise (SiN) recognition imply the additional recruitment of higher cognitive resources, such as attention and working memory, which are necessary to maintain or improve hearing and speech perception in adverse listening conditions (Schmitt et al. 2022; Giroud et al. 2021).

Based on the fact that tinnitus and hearing loss commonly evolve as parts of the same syndrome (Davis and El Rafaie 2000), in the present neuroanatomical study, we used SBM in association with a ROI-based approach, and compared multiple gray matter indices (CT, CSA and CV) between two groups of almost perfectly matched older participants with and without tinnitus, both characterized by pure-tone hearing loss. A crucial aspect of our work is that we took into account the multidimensional facets of hearing, and harmonized the two groups not only in terms of pure-tone hearing but also of supra-threshold hearing parameters that can be measured using TC, FS und SiN tasks (Giroud et al. 2018; Lecluyse et al. 2013; Schmitt et al. 2022). Such a procedure is required to control for confounding effects, because older individuals commonly exhibit heterogenic atrophic profiles (Bethlehem et al. 2022) which are often manifested in poor performance on measures of auditory processing (Fullgrabe 2013; Fullgrabe et al. 2015; Hopkins and Moore 2011; Moore et al. 2014; Pichora-Fuller and Souza 2003), or in speech comprehension difficulties in adverse listening situations despite a pure-tone hearing threshold in the normative range (Fulton et al. 2015). The pre-selected ROIs included brain areas which have previously been proposed to contribute to the emergence and maintenance of tinnitus, tinnitus distress, or to be generally involved in higher order auditory processing (Elmer et al. 2014; Giroud et al. 2018; Kleinjung and Langguth 2020; Meyer et al. 2016; Muhlau et al. 2006; Profant et al. 2020; Rauschecker et al. 2010; Schmidt et al. 2018; Schneider et al. 2009). With this purpose in mind, we specifically focused on the bilateral superior temporal gyrus (STG), Heschl’s gyrus (HG), transverse temporal sulcus (TTS), planum temporale (PT), planum polare (PP), superior temporal sulcus (STS), insula, angular (AG) and supramarginal gyrus (SMG), precuneus, inferior frontal gyrus (IFG, pars opercularis, triangularis and orbitalis), prefrontal cortex (PFC), cingulate cortex (CC), parahippocampal gyrus (PHG), hippocampus and on the amygdala. Furthermore, within the tinnitus group we examined possible associations between gray matter peculiarities, tinnitus duration and distress using assumption-free multiple linear regression analyses that took into account all vertices of the pre-selected ROIs.

Most of the ROIs examined in our work are also reconcilable with current dual-stream models of auditory processing across species (Hickok and Poeppel 2007; Rauschecker and Scott 2009). In fact, results from both humans and monkeys leave little room for doubt about the fundamental role of the ventral stream in decoding spectrally complex sounds and recognizing auditory objects (Rauschecker 2012), whereas the dorsal stream contributes to spatial processes, sensory-motor integration as well as to higher order auditory cognition (Hickok and Poeppel 2007; Rauschecker 2012; Rauschecker and Scott 2009). The neural underpinnings of auditory object recognition and higher order auditory processing also partially overlap across different domains, including speech, music, animal vocalizations and environmental sound recognition (Hickok and Poeppel 2007; Lewis et al. 2004; Rauschecker 2012; Rauschecker and Scott 2009). Hence, it is conceivable to assume that tinnitus may involve hard-wired evolutionary circuits that are additionally shaped by ecological adaptations or external influences, even though this perspective is controversial.

Since tinnitus is closely related to the perception of phantom sounds, we expected to find anatomical between-group differences in primary and associative auditory areas situated in the temporal lobe (Aldhafeeri et al. 2012; Allan et al. 2016; Boyen et al. 2013). Furthermore, based on previous studies indicating a contribution of extra-auditory areas to the maintenance of tinnitus, we predicted that the TIHL group would also be characterized by a differential gray matter architecture in the frontal (Aldhafeeri et al. 2012; Allan et al. 2016; Husain et al. 2011) and parietal (Adjamian et al. 2014; Allan et al. 2016; Meyer et al. 2016) cortex as well as in limbic areas such as the amygdala and hippocampus (Besteher et al. 2019; Landgrebe et al. 2009; Profant et al. 2020; Rauschecker et al. 2010; Tae et al. 2018). However, due to the heterogeneity of previous results, we did not have clear a-priori hypotheses as to whether individuals affected by tinnitus will exhibit increased (Boyen et al. 2013; Husain et al. 2011; Muhlau et al. 2006) or decreased (Aldhafeeri et al. 2012; Allan et al. 2016; Besteher et al. 2019; Landgrebe et al. 2009; Leaver et al. 2012) gray matter parameters in the examined brain areas.

## Materials and methods

### Participants

**Fig. 1:**
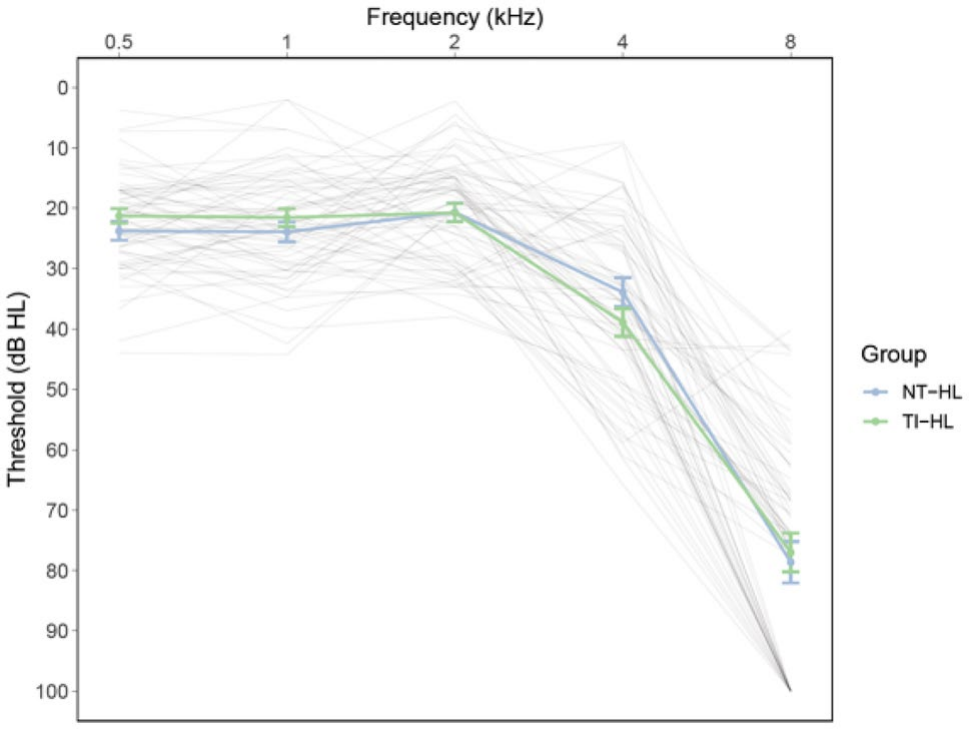
Pure-tone audiometric profiles. Pure-tone hearing thresholds (dB HL) for the frequencies in the range of 0.5–8 kHz are depicted separately for the tinnitus group with hearing loss (TIHL, green line) and the non-tinnitus group with hearing loss (NTHL, blue line). Each gray line represents an individual participant. *dB* decibel, *HL* hearing loss

For the present study, we recruited two groups of older participants in the age-range of 63–78 years with (tinnitus and hearing loss, TIHL, *N* = 33, mean age = 70.51, SD = 3.84) and without (non-tinnitus and hearing loss, NTHL, *N* = 33, mean age = 69.21, SD = 3.31) tinnitus, but both characterized by mild to moderate pure-tone hearing impairment which was reflected in pure-tone averages (PTA) between 25 and 60 dB hearing loss (Fig. 1). The two groups were matched for sample size, age, sex, handedness, years of education as well as for the degree of pure-tone and supra-threshold hearing loss (i.e., frequency selectivity and temporal compression) as well as for SiN recognition. Furthermore, the two groups demonstrated comparable cognitive capabilities in terms of fluid and crystallized intelligence, processing speed and verbal fluency. The participants of the TIHL group exhibited chronic symptoms for at least 2 years, with a mean tinnitus duration of 19.34 years (SD = 13.21). Furthermore, in the TIHL group, distress was assessed using the tinnitus handicap inventory (THI) (Newman et al. 1996) as well as the tinnitus questionnaire (TQ) (Goebel and Hiller 1994). The demographic, psychometric and audiometric data of the participants are summarized in Table 1.

**Table 1:** Overview of the demographic, psychometric and audiometric data of the TIHL (tinnitus and hearing loss) and NTHL (non-tinnitus and hearing loss) groups

Variables	Test/questionnaire	TIHL	NTHL	*t* value	*p* value
Age (years)		69.21 ± 3.31	70.51 ± 3.84	1.47	0.14
Sex (binary)		18 m 15 f	17 m 16 f	0.24	0.80
Education (years)		14.75 ± 3.15	14.18 ± 3.13	0.73	0.46
Fluid intelligence	KAI	52.69 ± 9.61	54.28 ± 9.13	− 0.68	0.49
Crystallized intelligence	MWT-B	65.90 ± 9.98	65.73 ± 7.83	0.07	0.93
Processing speed	ZST-WAIS	47.67 ± 5.10	48.75 ± 6.09	− 0.77	0.44
Verbal fluency	LPS	59.00 ± 9.67	62.85 ± 10.91	− 1.51	0.13
Mean pure-tone hearing threshold (dB hearing loss)	Pure-tone audiometry	35.91 ± 6.35	35.89 ± 6.27	− 0.01	0.93
Supra-threshold hearing	Temporal compression (TC)	4.07 ± 8.41	3.96 ± 7.88	− 0.05	0.95
	Frequency selectivity (FS)	16.58 ± 16.00	15.46 ± 19.60	− 0.25	0.80
Speech-in-noise (SiN) recognition (SNR)	Oldenburger sentence test (OLSA)	− 2.77 ± 2.10	− 3.27 ± 1.90	− 1.12	0.26
Tinnitus duration (years)	Self-report	19.34 ± 13.21			
Tinnitus distress	Tinnitus questionnaire (TQ)	20.55 ± 15.68			
	Tinnitus handicap inventory (THI)	19.61 ± 17.73			

Continuous variables are represented by the mean value plus/minus standard deviation

*KAI* Kurztest für Allgemeine Basisgrössen der Informationsverarbeitung, *MWT-B* Mehrfachwahl-Wortschatz-Intelligenztest, version B, *ZST* Zahlen-Symbol-Test of the Wechsler adult intelligence scale (WAIS), *LPS* Leistungsprüfsystem, *dB* decibel, *SNR* signal-to-noise ratio

All participants were native Swiss-German speakers, consistently right-handed (Annett 1970), and demonstrated a score of at least 26 points on the Montreal Cognitive Assessment (MoCA) scale (Nasreddine et al. 2005). None of the participants reported a history of neurological, psychiatric or developmental language disorders, took psychotropic medication, or wore hearing aids. Furthermore, due to possible confounds simultaneous and early bilinguals (Abutalebi and Green 2007; Rodriguez-Fornells et al. 2009) and well as individuals with more than 6 h of music practice per week were excluded (Jancke 2009). The participants were paid for participation, and gave informed written consent in accordance with the procedures of the local ethics committee (Kantonale Ethikkommission Zürich, ethics application number BASEC-NR, 2017-00284) and the declaration of Helsinki.

### Cognitive capabilities

To ensure that the two groups exhibited comparable general cognitive capabilities, we tested a specific set of psychometric variables, namely fluid and crystallized intelligence, processing speed and phonemic verbal fluency. Fluid intelligence was examined using the KAI test (‘Kurztest für Allgemeine Basisgrössen der Informationsverarbeitung’) (Lehrl 1992). This procedure consisted of reading aloud meaningless sequences of 20 letters as quickly as possible, and of repeating auditory-presented letters and digits of increasing length (up to nine items). Otherwise, crystallized intelligence was assessed using the MWT-B test (‘Mehrfachwahl-Wortschatz-Intelligenztest’, version B) (Lehrl 1977) which included 37 trials ordered as a function of difficulty level, and the participants had to select the unique word with a meaning from a set of five words that included four pseudowords. Both the KAI and MWT-B tests enable to estimate general intelligence in a short time, and have previously been shown to correlate fairly well (*r* ~ 0.7) with the global intelligence quotient in healthy adults (Lehrl 1992). Furthermore, processing speed was screened by means of the ZST (‘Zahlen-Symbol-Test’) test which is included in the Wechsler adult intelligence scale (WAIS) (Wechsler 1981). This classical digit-symbol-coding test is a speed-dependent procedure in which simple graphical symbols have to be associated with predetermined numbers in the range of 1–9 as quickly as possible according to an assignment key printed on the test sheet. The test value is calculated based on the number of symbols that are correctly paired with the corresponding numbers in the time range of 120 s. Finally, phonemic verbal fluency was evaluated using the LPS (‘Leistungsprüfsystem’), and consisted of generating as many words as possible beginning with the phonemes /f/, /k/ or /r/ in the time limit of one minute (Horn 1962). Phonemic verbal fluency has been proven to be particularly effective to assess frontal lobe functions (Henry and Crawford 2004). All the four psychometric tests were analyzed and compared between the two groups according to normative T-values.

### Hearing and speech-in-noise recognition

To examine participants’ pure-tone and supra-threshold hearing capabilities, we applied established screening procedures that have already been used and described in detail in earlier studies (Giroud et al. 2018; Lecluyse et al. 2013; Wagener et al. 1999). The comprehensive hearing assessment was conducted in a sound booth, and the acoustic stimuli were presented using a Genelec loudspeaker (8030B) positioned at 0° azimuth and at 1.5 m from the subjects’ head. In a first step, we objectified absolute pure-tone thresholds as a correlate of peripheral hearing acuity. According to this procedure, which consisted of a probe-detection paradigm with 250 ms pure-tones presented at 500, 1000, 2000, 4000 and 8000 Hz (Lecluyse et al. 2013), all participants demonstrated pure-tone thresholds of maximal 60 dB hearing loss averaged across the octave frequencies between 500 and 8000 Hz (Fig. 1). In particular, the PTA (averaged over all 5 tested frequencies) was of 35.89 dB hearing loss for the NTHL group (SD = 6.27) and of 35.91 dB for the TIHL group (SD = 6.35).

In a next step, we collected additional hearing estimates using two supra-threshold measurements, namely FS and TC, in association with a forward-masking paradigm (Lecluyse and Meddis 2009; Lecluyse et al. 2013). Forward masking occurs when a probe sound cannot be perceived due to the presence of a preceding masker sound, and such a paradigm is commonly used to identify the masked threshold, which corresponds to the quietest masking tone level still capable of preventing the detection of the probe tone (Lecluyse and Meddis 2009; Lecluyse et al. 2013). In our case, the signal level of the probe tone was fixed, whereas the masker level varied adaptively. The FS task consisted of a masker tone of 108 ms which was followed, after a delay of 10 ms, by a 16 ms probe tone presented at 10 dB above the individual absolute hearing threshold. The participants were asked to determine via button press whether they heard the probe tone or not. Importantly, the intensity level of the masker tone varied according to an adaptive algorithm, and its loudness level either increased or decreased in steps of 2 dB as a function of individuals’ accuracy. This procedure was applied to five probe frequencies (500, 1000, 2000, 4000 and 8000 Hz), and the loudness ratio between the masker and probe tones varied in a predetermined manner (0.7:1, 0.9:1, 1:1, 1.1:1, 1.3:1). Furthermore, to draw attention, each trial was signalized by a cue tone that was presented 500 ms before the masker. For statistical analyses, we calculated the depth between the masker levels at the highest and lowest frequencies, and averaged these values across the five probe frequencies. Such an approach has been shown to provide a valid measure for assessing FS in both normal hearing and hearing-impaired participants (Lecluyse et al. 2013). In addition, TC abilities were measured using the same adaptive forward-masking paradigm with five masker frequencies (500, 1000, 2000, 4000 and 8000 Hz). However, the main difference was that in the TC task the gap between the masker and the probe tones varied adaptively in steps of 10, 30, 50, and 70 ms. The steepness of the psychometric slopes as a function of gap duration were averaged to obtain an objective measure of TC (Lecluyse et al. 2013). Both the FS and TC task were implemented using the MATLAB software.

Finally, as an additional supra-threshold measurement, SiN recognition was assessed using an established task, namely the Oldenburger sentence test (OLSA) (Wagener et al. 1999). This procedure enables to measure the signal-to-noise ratio (SNR) at which a participant is able to correctly reproduce 50% of the words of a sentence embedded in speech-shaped background white noise. Both sentences and noise were initially presented at 65 dB sound pressure level (SPL), and the sentence SPL varied adaptively after each response to assess the SNR. Since the test material consisted of low-constraint sentences, it is unlikely that the participants were able to infer possible candidate words from the context. The auditory stimuli of the SiN task were presented using the MACarena software (https://shorturl.at/ckry7).

### MRI data acquisition and surface-based morphometry

High-resolution T1-weighted images were obtained for each participant by means of a 3 Tesla Philips Ingenia scanner (Philips Medical Systems, Best, The Netherlands) equipped with a 12-channel head coil. The three-dimensional anatomical images were acquired using a Turbo-Field-Echo sequence with the following parameters: echo time = 3.7 ms, repetition time = 8.2 ms, field of view = 240 × 240 × 160 mm, acquisition matrix = 240 × 240, 160 slices per volume, isotropic voxel size = 1 × 1 × 1 mm, flip angle (α) = 90°, total scan time = 6 min. No participant was excluded due to inhomogeneous MRI data.

Since the SBM analyses have been performed according to standard procedures and were almost the same as those previously used by our groups (Elmer et al. 2013, 2014; Giroud et al. 2018), some text passages were reiterated from our previous studies. Cortical surface reconstruction was performed with the FreeSurfer image analysis suite (version 7.3.2), which is freely available and documented online (http://freesurfer.net/). The SBM analyses implemented in the FreeSurfer pipeline involved several preprocessing steps, which have already been extensively described in prior publications (Dale et al. 1999; Fischl and Dale 2000; Fischl et al. 1999a, b, 2001, 2002, 2004a, b; Segonne et al. 2004). Briefly, the three-dimensional structural T1-weighted MRI scans were used to construct models of each subject's cortical surface and to measure brain features such as CT, CSA and CV. CT is defined as the minimal distance between gray-white matter borders and the pial surface at each vertex of the tessellated surface (Fischl and Dale 2000). Otherwise, CSA is specified as the mean area of the region at the respective vertex, while CV is simply the arithmetic product of CT and CSA. The FreeSurfer procedure is fully automated and involves segmentation of the cortical white matter (WM) (Dale et al. 1999), tessellation of the gray/white matter junction, inflation of the folded surface tessellation patterns (Fischl et al. 1999a, b), and automatic correction of topological defects in the resulting manifold (Fischl et al. 2001). This surface model was then used as the starting point for a deformable surface algorithm designed to find the gray/white and pial (gray matter/cerebrospinal fluid) surfaces with sub-millimeter precision (Fischl and Dale 2000). Both histological analyses (Rosas et al. 2002) and manual measurements (Kuperberg et al. 2003; Salat et al. 2004) have previously been used to validate the procedure for measuring CT. The method uses both intensity and continuity information from the surfaces in the deformation procedure to be able to interpolate surface information for regions in which the MRI image is ambiguous (Fischl and Dale 2000). For each subject, CT of the cortical ribbon was computed on a uniform grid (comprised by vertices) with 1 mm spacing across both cortical hemispheres, with the thickness being defined by the shortest distance between the gray/white and pial surface models. The CT maps produced are not limited to the voxel resolution of the images and are thus sensitive to detecting sub-millimeter differences between groups (Fischl and Dale 2000). CT measures were mapped onto the inflated surface of each participant’s brain reconstruction, allowing the visualization of the data across the entire cortical surface (i.e., gyri and sulci) without being obscured by the cortical folding pattern. Each subject’s reconstructed brain was then morphed to an average spherical surface representation that optimally aligned sulcal and gyral features across subjects (Fischl et al. 1999a, b). This procedure provides an accurate matching of morphologically homologous cortical locations among participants on the basis of each individual’s anatomy while minimizing metric distortions. This transform was used to map CT and CSA measurements into a common spherical coordinate system. In addition, a parcellation of the cerebral cortex into units based on gyral and sulcal structures was performed (Desikan et al. 2006; Fischl et al. 2004b), and a variety of surface-based data including maps of CT and CSA were created. For all subjects, the data were re-sampled into a common spherical coordinate system (Fischl et al. 1999a, b). The CT and CSA maps were then smoothed on the surface tessellation using an iterative nearest-neighbor averaging procedure (50 iterations were used, which is equivalent to applying a two-dimensional Gaussian smoothing kernel along the cortical surface with a full-width at half-maximum of about 10 mm). These maps were then subjected to group comparisons.

### Pre-selection of the ROIs

**Fig. 2:**
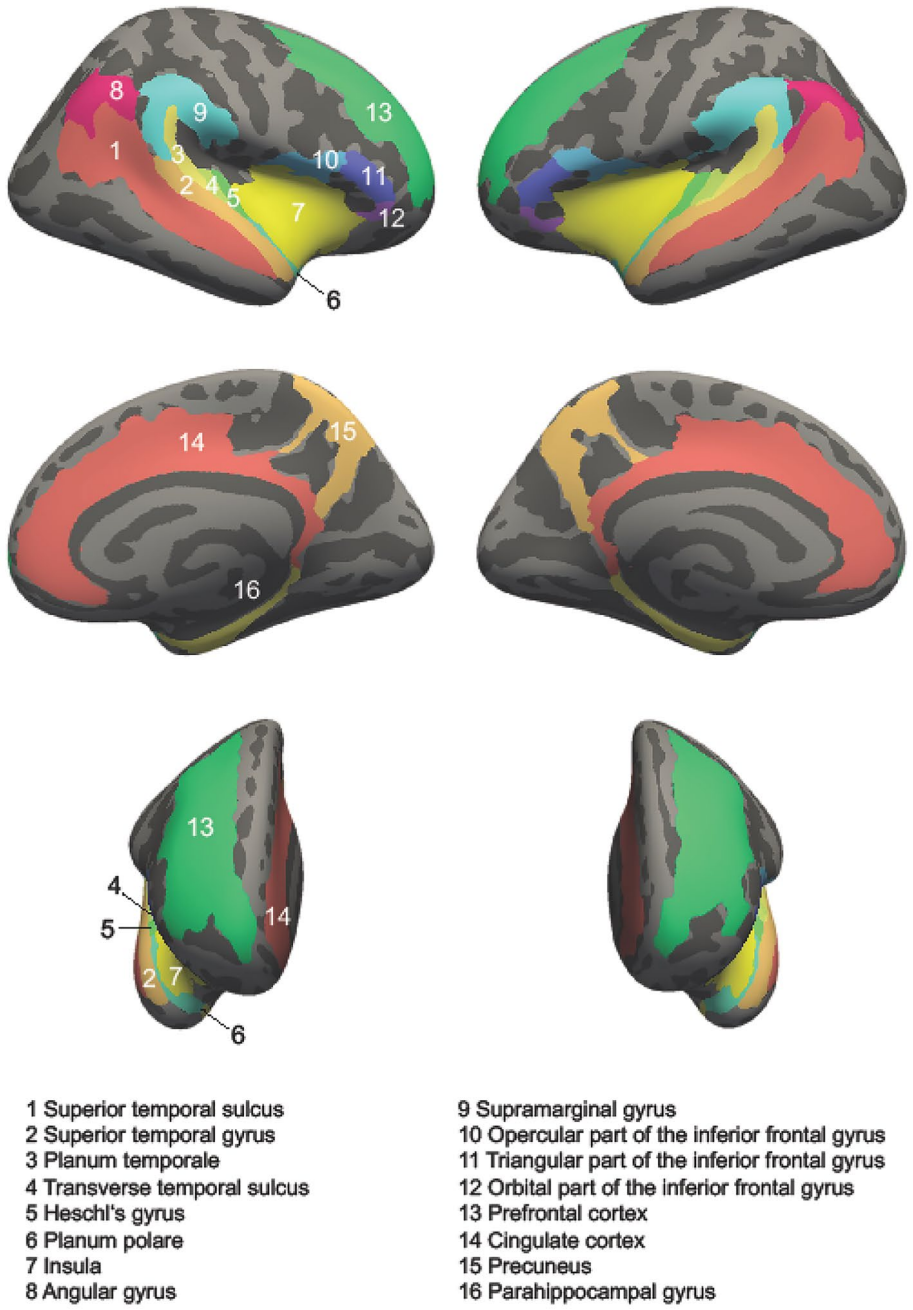
Sixteen out of 18 regions-of-interest (ROIs) used for the morphometric analyses (without hippocampus and amygdala). First row = lateral view, second row = medial view, third row = anterior view. Left column = right hemisphere, right column = left hemisphere. The number 1–16 represent the 16 regions-of-interest without hippocampus and amygdala

**Fig. 3:**
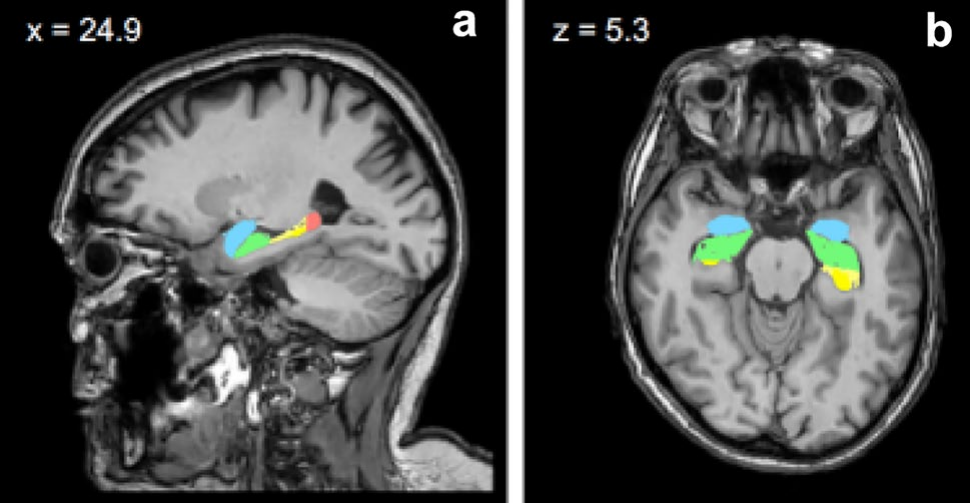
Depiction of the two additional subcortical regions-of-interest (ROIs) included in the group comparisons, namely amygdala and hippocampus. **a** = sagittal view, **b** = horizontal view, light blue = amygdala, green = head of the hippocampus, yellow = body of the hippocampus, pink = tail of the hippocampus

In the present work, we opted for a statistically more lenient ROI approach to maximize statistical power instead of using conservative whole-brain analyses because we did not expect to find relevant group differences beyond the selected regions. The cortex was parcellated into a-priori defined sub-regions according to the aparc.a2009s annotation (Destrieux et al. 2010), and CT, CSA and CV were extracted from 16 bilateral ROIs (Fig. 2), including the CC as well brain areas situated in the frontal (IFG pars opercularis, pars triangularis, pars orbitalis and PFC), the temporal (STG, HG, TTS, PT, PP, STS, insula, PHG) and the parietal (precuneus, AG and SMG) lobes. In addition, we also evaluated the volumes of the hippocampus and the amygdala (Fig. 3), resulting in a total of 18 target ROIs. Thereby, it is noteworthy to mention that instead of evaluating the hippocampus on a gross anatomical scale, we separately examined the volumes of three sub-regions, namely hippocampal head, body and tail (Wisse et al. 2014) using the algorithm by Iglesias et al. (Iglesias et al. 2015) as well as the amygdala (Saygin et al. 2017). The selected brain regions were chosen based on previous work that identified them as relevant for explaining the origin, the maintenance or the emotional distress of tinnitus (Kleinjung and Langguth 2020; Meyer et al. 2016; Muhlau et al. 2006; Profant et al. 2020; Rauschecker et al. 2010; Schmidt et al. 2018; Schneider et al. 2009; Schecklmann et al. 2013).

### Statistical analyses

Before the main experiment, we used the G*power software to calculate the smallest sample size needed (Faul et al. 2007, 2009). According to this procedure, a mean effect size (partial eta = 0.06) related to the difference in slope between two distinct groups requires a minimum total sample size of *N* = 54. Since we included two groups of 33 participants each, our sample size was larger than the required minimum. The demographic, psychometric and audiometric data of the participants were evaluated using *t* tests for independent samples (two-tailed, Table 1) and the IBM SPSS Statistics 26 software package (SPSS, an IBM company, Armonk, New York, USA). The between-group comparison of amygdala and hippocampus (head, body and tail) volumes as well as the multiple linear regressions between these anatomical structures and tinnitus distress (THI)/duration were computed in R (version 4.0.3; R Core Team 2020). In contrast, the between-group comparisons of the 16 remaining cortical ROIs (without hippocampus and amygdala) as well as the corresponding multiple linear regression analyses were conducted in the FreeSurfer environment using the general linear model (GLM) procedure. Importantly, due to the high collinearity between THI and TQ scores (Pearson’s *r* = 0.851, *p* < 0.001, two-tailed), possible associations between anatomical parameters and tinnitus distress were only addressed using the THI which has been shown to provide a reliable measure of tinnitus-related emotional states (Newman et al. 1996).

#### Anatomical analyses: ROI-based group comparisons

For the amygdala and the hippocampal sub-regions, we only examined between-group volume differences by means of separate multiple linear regressions analyses with the factors group and volume, whereas intracranial volume (Z-standardized) was treated as a covariates of no interest. In contrast, the remaining 16 cortical ROIs were compared vertex-wise (CT, CSA and CV) between the two groups. In particular, after having fitted the GLM with the built-in function implemented in FreeSurfer, we ran 10,000 Monte Carlo z-simulations to correct for multiple comparisons at the cluster level (Nichols and Hayasaka 2003) using a vertex-wise/cluster-forming threshold of 0.05 and a cluster-wise *p* value of 0.05 (Bonferroni-corrected for analyzing both hemispheres). Since the two groups were almost perfectly matched, for all FreeSurfer analyses we did not model any covariates.

#### Multiple linear regression analyses: brain–tinnitus relationships

To circumvent the problem of circularity and to avoid double dipping (Kriegeskorte et al. 2009), we abstained from computing linear regression analyses by extracting the anatomical clusters that significantly differed between the two groups. Instead, we used an assumption-free procedure, and assessed linear relationships between all vertices included in the 16 ROIs (without hippocampus and amygdala) and THI scores/tinnitus duration. Additional multiple linear regressions were performed to inspect associations between amygdala and hippocampal volumes and the same two behavioral variables of interest. For all regression analyses computed within the TIHL group, sex, age and PTA were modeled as covariates of no interest. Furthermore, the regression analyses focusing on amygdala and hippocampal volumes additionally included intracranial volume as a covariate.

## Results

### Demographic, psychometric and audiometric data

The statistical analyses of the demographic, psychometric and audiometric data confirmed that the two groups were almost perfectly matched. In fact, we did not reveal significant between-group differences for age, sex, years of education, fluid and crystallized intelligence, processing speed and verbal fluency. In addition, the two groups were also comparable in terms of pure-tone hearing, supra-threshold hearing estimates and SiN performance (Table 1 and Fig. 1).

### Anatomical analyses: ROI-based group comparisons

**Fig. 4:**
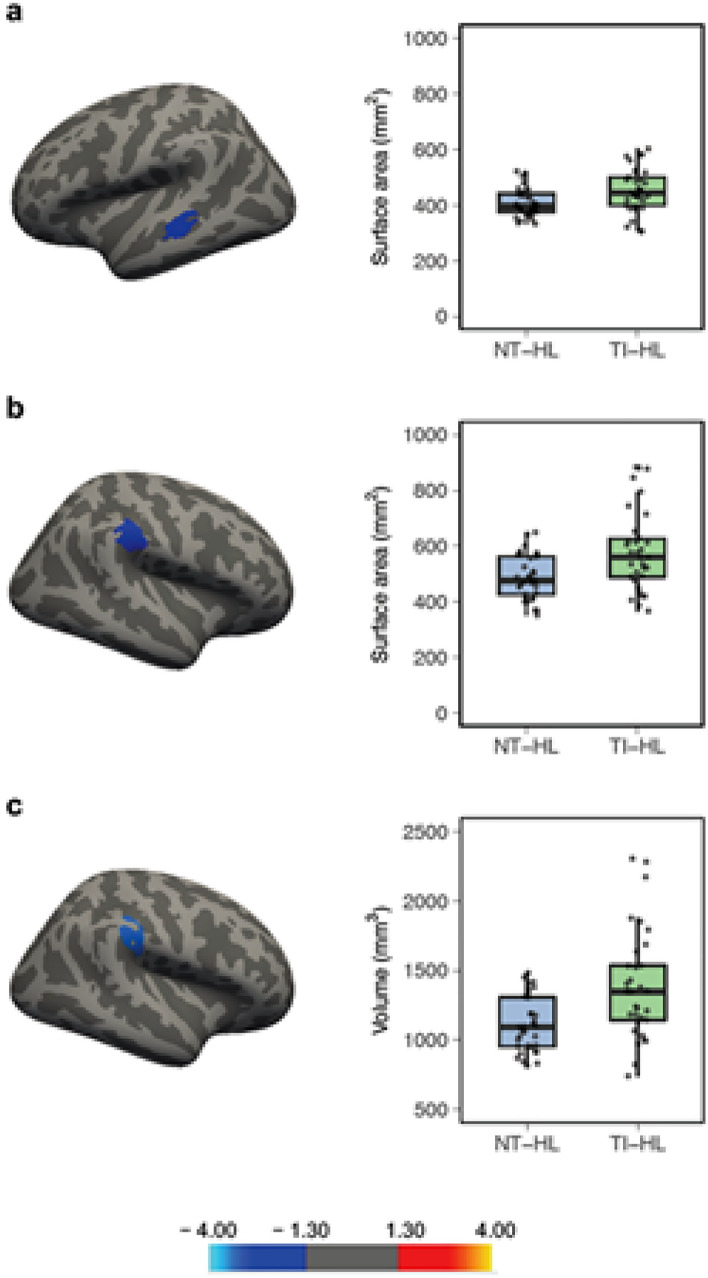
Significant ROI-based results of the vertex-wise comparisons computed in FreeSurfer (without amygdala and hippocampus) between the TIHL (tinnitus and hearing loss) and NTHL (non-tinnitus and hearing loss) groups. The boxplots on the right side depict the single subject data corresponding to the significant clusters, with median and interquartile range. **a** middle-anterior part of the left superior temporal sulcus (STS), **b** and **c** right supramarginal gyrus (SMG) and posterior part of the planum temporale (PT). Color scale = cluster-wise *p* value (− log10), blue color = TIHL > NTHL, red color = TIHL < NTHL

**Fig. 5:**
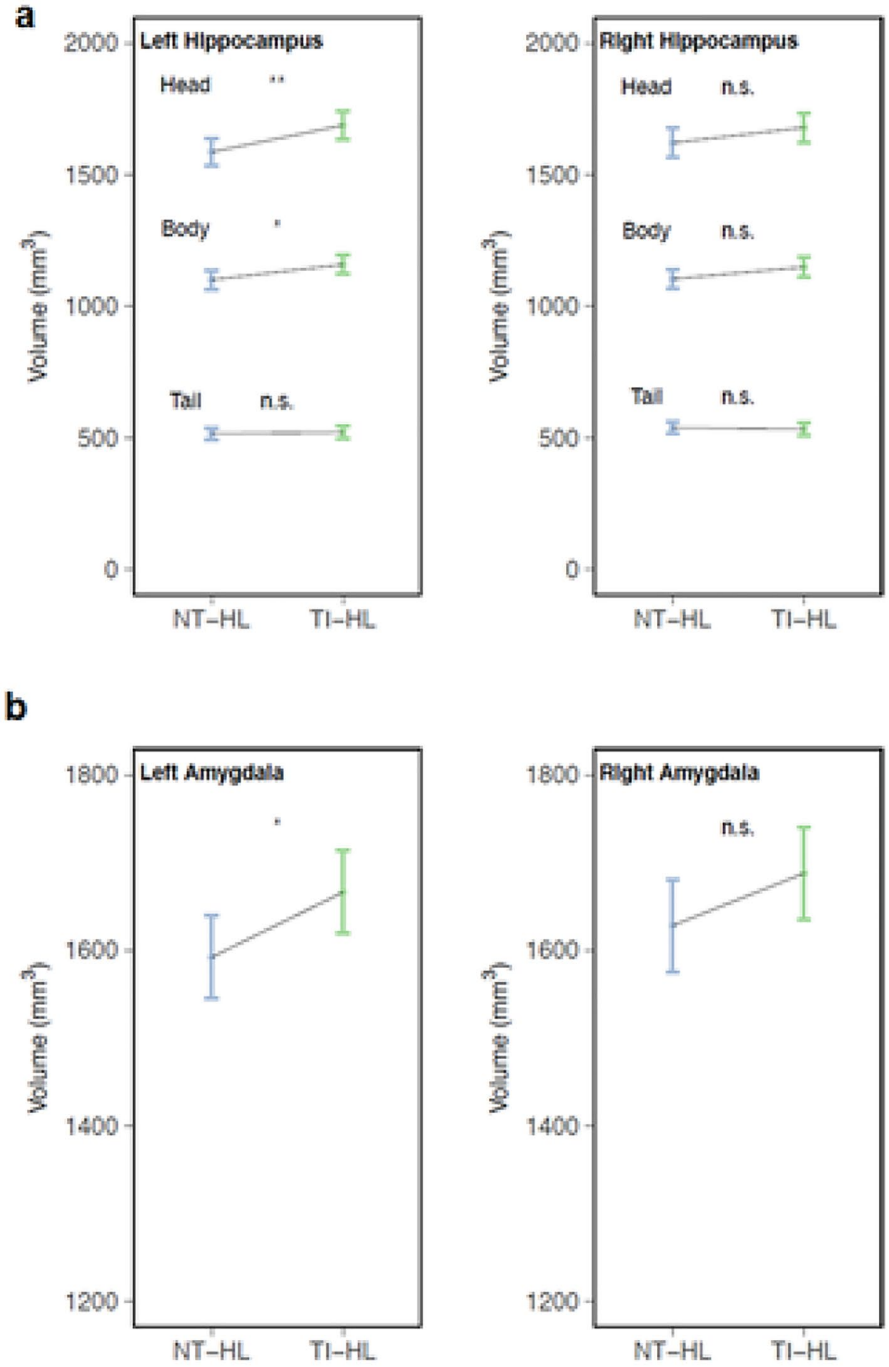
Volumetric results of the hippocampus (**a**, head, body and tail) and the amygdala (**b**), the error bars indicate standard deviation. The volumetric results are shown based on the model prediction values that took into account the influence of intracranial volume (fitted as a covariate of no interest in the multiple linear regressions). *NTHL* non-tinnitus and hearing loss, *TIHL* tinnitus and hearing loss. * *p* < .05, ** *p* < .01. *n.s* = not significant

The vertex-wise group comparisons of CT, CSA and CV (16 ROIs without amygdala and hippocampus) revealed increased CV and CSA of the right SMG and the posterior part of the right PT in the TIHL compared to the NTHL group (Fig. 4b, c, Table 2). Furthermore, the TIHL group exhibited a larger CSA in a cluster situated in the middle-anterior part of the left STS (Fig. 4a and Table 2). Otherwise, the multiple linear regression analyses used to compare amygdala and hippocampal (head, body and tail) volumes between the two groups yielded significant results in left-hemispheric structures only. In particular, the TIHL group demonstrated an increased gray matter volume of the amygdala (NTHL vs. TIHL, *β* = − 74.09, CI = − 141.52/− 6.67, *p* = 0.032) as well as of the head (NTHL vs. TIHL, *β* = − 101.30, CI = − 176.05/− 26.54, *p* = 0.009) and body (NTHL vs. TIHL, *β* = − 57.76, CI = − 108.67/− 6.85, *p* = 0.027) of the hippocampus (Fig. 5a, b).

**Table 2:** Significant between-group differences in the ROI-based analyses performed with FreeSurfer (without amygdala and hippocampus)

Measure	Brain region	Maximal value	Size (mm^2^)	MNI coordinates			CWP
				X	X	Z	
LH area	Superior temporal sulcus	− 2.43	398.9	− 49.1	− 32.8	− 1.7	0.024
RH area	Supramarginal gyrus and planum temporale	− 3.99	543.59	57.1	− 27.5	29.3	0.003
RH volume	Supramarginal gyrus and planum temporale	− 3.64	456.46	57.3	− 30.2	36.6	0.001

The TIHL group consistently showed increased anatomical values compared to the NTHL group

*LH* left hemisphere, *RH* right hemisphere, *MNI* Montreal neurological institute, *CWP* cluster-wise *p* value

### Multiple linear regression analyses: brain–tinnitus relationships

**Fig. 6:**
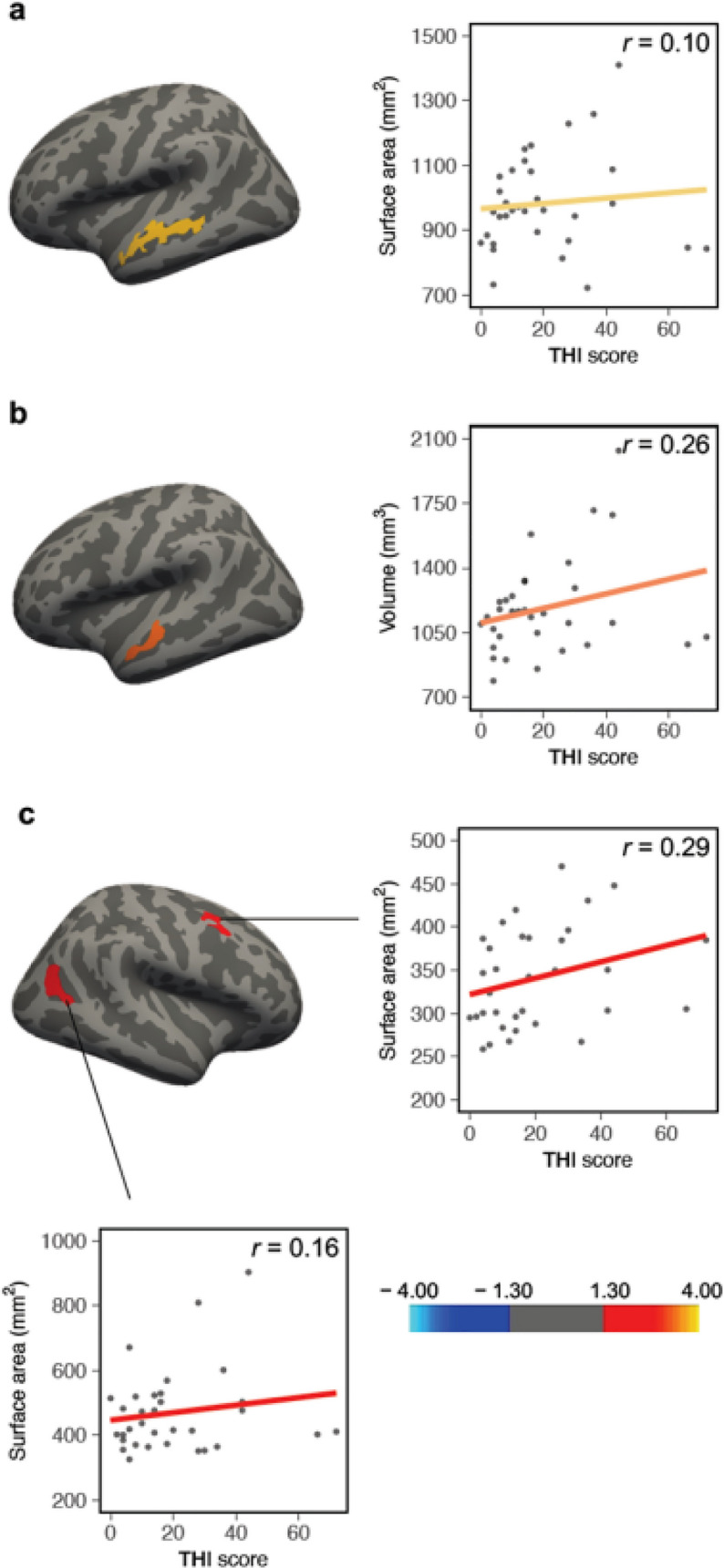
Significant positive relationships within the TIHL (tinnitus and hearing loss) group between gray matter indices and tinnitus distress (THI) in the ROI-based vertex-wise multiple linear regression analyses. **a** and **c** cortical surface area, **b** cortical volume. Color scale = cluster-wise *p* value (− log10), red = positive association, blue = negative association

**Fig. 7:**
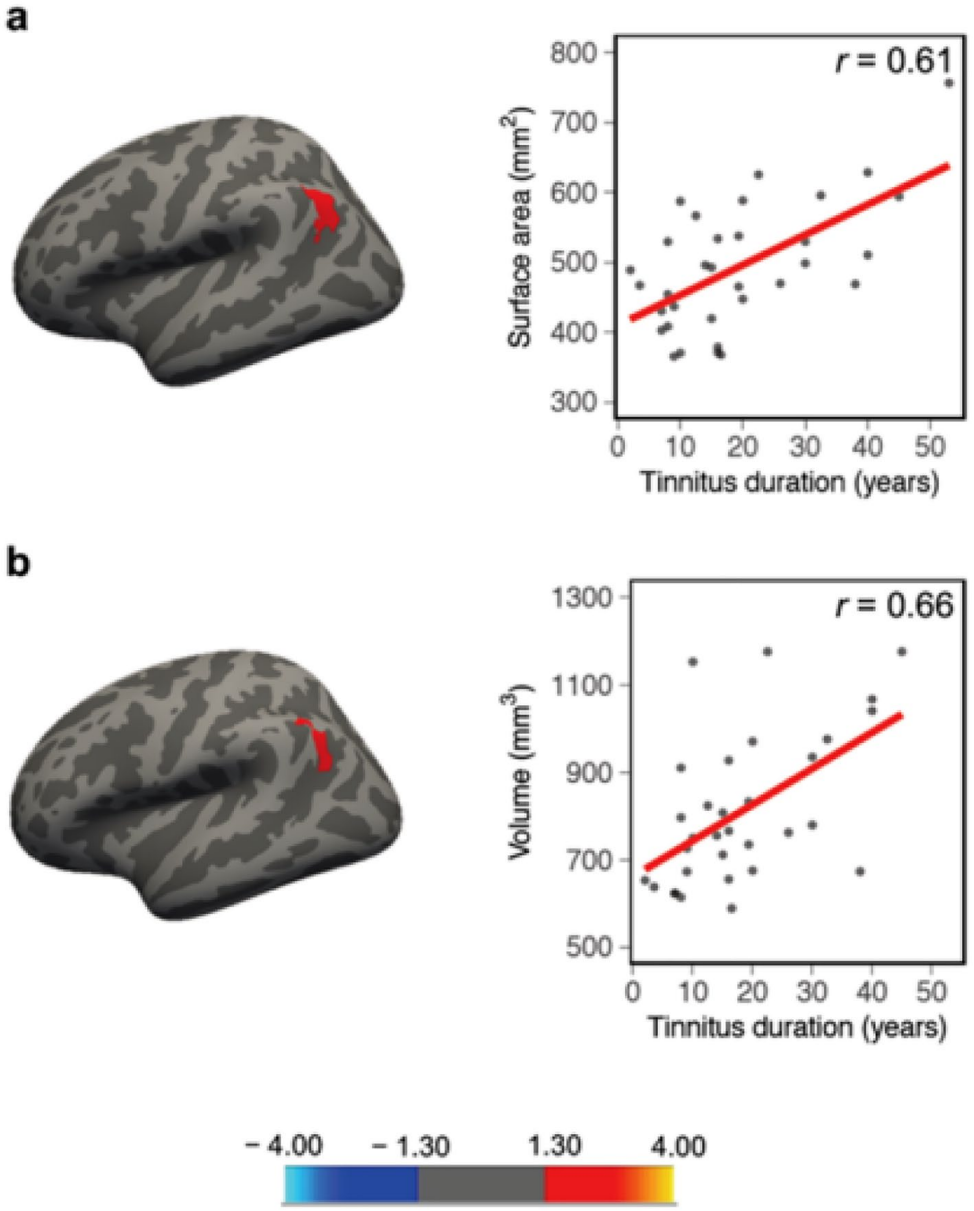
ROI-based vertex-wise multiple linear regression analyses, significant positive relationships within the TIHL (tinnitus and hearing loss) group between gray matter indices and tinnitus duration (years). **a** cortical surface area, **b** cortical volume. Color scale = cluster-wise *p* value (− log10), red = positive association, blue = negative association

Within the TIHL group, we additionally performed ROI-based (16 ROIs without amygdala and hippocampus) and vertex-wise multiple linear regressions to inspect possible associations between gray matter indices, tinnitus distress (THI) and tinnitus duration (years). According to these analyses, CSA (Fig. 6a and Table 3) as well as CV (Fig. 6b and Table 3) of the left middle-anterior STS and STG were positively related to tinnitus distress. Furthermore, we found a positive association between CSA and THI scores of two clusters situated in the right PFC and posterior STS (Fig. 6c and Table 3). Further multiple linear regressions performed to uncover relationships between gray matter parameters and tinnitus duration revealed positive associations for CSA (Fig. 7a and Table 3) and CV (Fig. 7b and Table 3) in two regions located in the left posterior STS and AG. In contrast, none of the separate multiple linear regression analyses used to inspect volumetric associations between amygdala and hippocampal volumes and tinnitus distress/duration reached significance (all *p* values > 0.05).

**Table 3:** Significant ROI-based vertex-wise multiple linear regressions within the TIHL (tinnitus and hearing loss) group for the positive relationships between gray matter parameters, tinnitus distress (THI) and tinnitus duration (years)

Relationships	Measure	Brain region	Maximal value	Size (mm^2^)	MNI coordinates			CWP
					X	X	Z	
Gray matter–tinnitus distress (THI)	LH area	Superior temporal sulcus and superior temporal gyrus	3.74	918.33	− 46.5	− 35	− 4.5	0.0002
	LH volume	Superior temporal sulcus and superior temporal gyrus	5.05	412.1	− 49.3	− 5.1	− 20.3	0.001
	RH area	Prefrontal cortex	3.12	401.15	30.6	5.2	53.3	0.025
	RH area	Superior temporal sulcus	3.66	398.62	42.1	− 72.5	18.6	0.026
Gray matter–tinnitus duration (years)	LH area	Angular gyrus and superior temporal sulcus	4.87	469.35	− 41.4	− 60.8	30.7	0.003
	LH volume	Angular gyrus and superior temporal sulcus	3.16	412.1	− 38.8	− 57.9	25.4	0.006

*LH* left hemisphere, *RH* right hemisphere, *MNI* Montreal neurological institute, *CWP* cluster-wise *p* value

## Discussion

### General discussion

The present neuroanatomical study was an attempt to pave the way toward a better understanding of tinnitus as a syndrome characterized by the joint manifestation of auditory phantom sensations and hearing loss. Moreover, we took into account the multifaceted dimensions of hearing and speech processing in adverse listening conditions, and harmonized the two experimental groups not only in terms of hearing thresholds but also of supra-threshold hearing and SiN recognition. The TIHL and NTHL groups were also matched for several critical variables, such as sample size, age, sex, handedness, years of education as well as for general cognitive capabilities. Within this framework, the intertwining of tinnitus and hearing loss was manifested in gray matter peculiarities of perisylvian brain regions, including the right SMG (increased CV and CSA), the right posterior PT (increased CV and CSA) as well as the left middle-anterior part of the STS (increased CSA). In addition, the TIHL group demonstrated larger gray matter volumes of the left amygdala and of the left head and body of the hippocampus. Notably, the examination of brain–tinnitus relationships also brought to light multiple cortical ribbons positively related to tinnitus duration and distress. In particular, CSA of the left middle-anterior STS/STG was predictive of tinnitus distress, and this cluster overlapped with a similar region that turned out to be significant in the group comparison. Tinnitus distress also positively correlated with CV of the left anterior STS/STG, with CSA of the right dorsal PFC as well as with CSA of the right posterior STS. In contrast, tinnitus duration was positively associated with CSA and CV of the right AG and posterior part of the right STS.

### Regional morphometric group differences and brain–behavior relationships

#### Temporal lobe

Using a ROI-based morphometric approach, we were able to replicate a common finding in the tinnitus literature (Boyen et al. 2013; Husain et al. 2011; Landgrebe et al. 2009; Meyer et al. 2016; Schecklmann et al. 2013; Schneider et al. 2009), namely an altered gray matter architecture of the auditory-related cortex, which was manifested in increased CV and CSA of the right posterior part of the PT. Although little is known about the intrinsic meaning of different gray matter parameters, CV can be understood as a gross anatomical dimension that is influenced by the amount and size of neurons, dendrites and glial cells, whereas CSA may reflect cortical folding, gyrification, or even the number and width of cortical microcolumns (Rakic 1995, 2000; van der Meer and Kaufmann 2022; Zilles et al. 1988). However, since the microcolumnar organization of the auditory cortex as well as cortical folding patterns are mostly determined prenatally (Rakic 1995, 2000), it is questionable whether the increased CSA we uncovered in the right PT of the TIHL group can be viewed as a consequence of maladaptive cortical reorganization rather than reflecting anatomical predisposition. Further doubts arise from several studies which examined training-related plasticity across multiple domains, and commonly identified CT or CV, but not mandatorily CSA, as the most malleable gray matter indices (Elmer et al. 2014; Habibi et al. 2018; Langer et al. 2012; Woollett and Maguire 2011). Therefore, our data are not conclusive as to whether the gray matter parameters of the PT reflected either an innate susceptibility to develop tinnitus or rather maladaptive cortical changes induced by environmental or physiological forces.

Interestingly, the increased CV and CSA of the PT were restricted to the right hemisphere, which is consistent with current theories postulating a spectro-temporal division of labor in the associative auditory cortex (Griffiths and Warren 2002; Poeppel 2003; Schonwiesner et al. 2005; Zatorre and Belin 2001). Actually, the PT has been proposed to behave as a computational hub (Griffiths and Warren 2002) that operates in a slightly asymmetric manner, with a preference of the left hemisphere for temporal acoustic analyses and of the right hemisphere for decoding spectral information (Griffiths and Warren 2002; Poeppel 2003; Zatorre and Belin 2001). Given that all participants suffered from pure-tone tinnitus, we may speculate that this distinct auditory phantom sensation was possibly grounded in altered gray matter characteristics of the right PT, which resulted in an over-representation of spectral tinnitus attributes. However, since CV and CSA parameters of this brain region did not correlate with tinnitus distress or symptoms duration, our data strengthen the argument that the gray matter architecture of the right PT constituted a fundamental prerequisite for perceiving tinnitus, whereas its negative connotation and time-dependent amplification were possibly more likely related to auxiliary neural networks. Nevertheless, such a contribution of non-primary auditory areas to vivid auditory sensations in the absence of sound sources in the environment has, for example, also been documented in a variety of auditory imagery studies (Bunzeck et al. 2005; Meyer et al. 2007; Tian et al. 2016). In fact, in contrast to the visual modality (Kosslyn et al. 2001), the imagination of speech, music or environmental sound has convincingly been shown to rely on associative but not primary auditory areas (Bunzeck et al. 2005; Meyer et al. 2007).

As a second morphometric marker, our results highlighted an association between the tinnitus syndrome and CSA of the left middle-anterior part of the STS. The STS is not only one of the longest sulci of the brain (Specht and Wigglesworth 2018), but also a multimodal structure involved in a variety of sensory and cognitive functions, such as phonetic processing, speech and voice perception, audiovisual integration, motion analyses as well as in the discernment of social cues (Belin et al. 2000; Hein and Knight 2008; Specht and Wigglesworth 2018). Interestingly, multimodal processes in the STS have also been shown to follow a gradient along its anterior–posterior axis, with functional activations related to introspection, biological motion and face perception restricted to the posterior portion, and voice, language and general auditory processing confined to the left middle-anterior part (Beauchamp 2015; Binder et al. 2000). Furthermore, lesions in the bilateral STS have been shown to provoke pure auditory agnosia, a condition in which patients are not more able to identify non-speech sounds such as whistling, coughing and crying despite preserved speech comprehension abilities (Clarke et al. 2000; Gutschalk et al. 2015). In addition, the STS has been linked to the categorization of tones (Schulze et al. 2009) and unfamiliar non-phonemic auditory patterns in general (Liebenthal et al. 2010). Such an involvement of the middle-anterior STS in domain-general auditory processing is not surprising, especially in light of previous work showing that the STS fulfills cyto- and receptor-architectonic properties common to sensory cortical fields (Morosan et al. 2005).

Importantly, the assumption-free multiple linear regression analyses also uncovered a positive relationship between CSA of the left middle-anterior STS/STG and tinnitus distress, and this cluster overlapped with a similar gray matter node that significantly differed between the two groups. Accordingly, our data emphasize that the gray matter infrastructure underlying chronic tinnitus can be part of the same matrix which is also involved in aversive or distressed reactions to tinnitus. Nevertheless, the precise functional role of the left middle-anterior STS in tinnitus distress remains purely elusive. One possible interpretation is that the anatomical peculiarities of this brain region were associated with a disturbance of the functional balance in the ventral processing stream mainly involved in auditory object recognition (Beauchamp et al. 2004; Werner and Noppeney 2010). In this vein, we may speculate whether such gray matter anomalies possibly reinforced the binding of tinnitus characteristics, or the maintenance of tinnitus in memory due to the misrepresentation of tone-specific auditory objects characterized by a distinctive sound quality and a defined spatial location (Griffiths and Warren 2004; Snyder and Alain 2007). This view would also be compatible with the idea that the between-group differences we revealed in the left middle-anterior part of the STS possibly reflected a change in tonotopic organization. In fact, even though a faithful topographic representation of frequencies is commonly attributed to the cochlea, to the subcortical nuclei, and to a lesser extent to the primary auditory cortex, there is evidence showing that tonotopic maps also co-exist in the STS (Cansino et al. 1994; Castro and Kandler 2010). The two perspectives sketched above would also be compatible with the additional results of the multiple linear regression analyses indicating that tinnitus distress correlated with CV in the left anterior STS/STG as well as with CSA in the right posterior STS.

#### Parietal lobe

The third main finding of our study was an increased CV and CSA of the right SMG in the TIHL compared to the NTHL group. This right-sided brain region, together with the AG, is part of the so-called temporo-parietal junction (TPJ) (Krall et al. 2015), plays a fundamental role in the orientation of attention toward new stimuli, and has been associated with contralateral neglect in both the visual and auditory modalities (Gutschalk and Dykstra 2015; Horiguchi et al. 2016; Karnath and Rorden 2012). The right TPJ has also been shown to be crucially involved in the processing of emotional information, and to contribute to emotional experiences (Dzafic et al. 2019; Grecucci et al. 2013; Zaitchik et al. 2010). According to the model proposed by Heller (1993), the right TPJ interacts with the prefrontal cortex to regulate emotional states through the modulation of valence and arousal. In particular, the theory postulates an asymmetry of emotional valence in the prefrontal cortex, with an involvement of the left hemisphere in positive and of the right hemisphere in negative emotion regulation. Furthermore, the right TPJ plays an important role in the guidance of arousal, which refers to a psychological state of alertness or excitation, for example, in association with stress, fear or anger (Messina et al. 2017). Based on this framework, we speculate that the increased CV and CSA of the right SMG in the TIHL group may have resulted in increased levels of arousal through the activation of the autonomic nervous system, while at the same time reducing the ability to ignore or filter out tinnitus-related information (McKenna et al. 2014). Although we did not reveal a positive relationship between anatomical indices of the right SMG and tinnitus characteristics, tinnitus duration was at least positively related to CSA and CV of the right AG. Furthermore, tinnitus distress positively correlated with CSA of the right dorsal PFC, possibly indicating that the right TPJ and the PFC are crucial components of a tinnitus-related valence-arousal network.

#### Amygdala and hippocampus

As a fourth major finding, the multiple linear regression models used to assess between-group differences in the amygdala and hippocampus only yielded left-hemispheric outcomes. In particular, the TIHL group exhibited an increased gray matter volume of the amygdala as well as of the head and body of the hippocampus. While anatomical changes in these two brain structures have already been documented in previous studies (Kraus and Canlon 2012; Profant et al. 2020; Zhang et al. 2019), the novel aspect of our work is that we provided a more comprehensive understanding of the hippocampal sub-regions related to tinnitus. Despite the fact that the hippocampus is classically considered the site of episodic and spatial memory formation (Scoville and Milner 1957; Squire and Zolamorgan 1991), recent perspectives also emphasized a fundamental role of this brain region in auditory cognition (Billig et al. 2022). Indeed, hippocampal responses to sounds have been observed in different domains, including passive exposure (Brown and Buchwald 1973), active listening (Kumar et al. 2014), and associative auditory learning (Derner et al. 2020). Furthermore, the hippocampus has been shown to support the binding of auditory features with disparate elements across both space and time (Olsen et al. 2012), and to be involved in speech (Urgolites et al. 2022) and music processing (Muller et al. 2013; Urgolites et al. 2022). Such a hippocampal contribution to multiple auditory domains is not entirely surprising, especially given its mutual connections with the auditory cortex (Billig et al. 2022). In this regard, both animal and human studies provided evidence for numerous functional and anatomical pathways from the auditory brainstem and the medial geniculate body to the hippocampus, and from there to auditory cortical core and belt areas (Jang and Choi 2022). Conversely, many circuits are also available for auditory cortex activity to reach the hippocampus, resulting in multiple reciprocal interactions (Billig et al. 2022).

Interestingly, even if the organization of the hippocampus along its longitudinal axis is somewhat controversial (Genon et al. 2021), several neuroimaging findings postulated the existence of functional sub-regions (Daugherty et al. 2016) which are legitimated by distinct functional and structural connectivity profiles (Adnan et al. 2016). Principally, the anterior part of the hippocampus, including head and body, has substantial connections with the amygdala, lateral temporal regions, the STG, the temporal pole, the insula as well as with the anterior CC and ventromedial PFC via the fornix-mammillary body-thalamic system and the uncinate fasciculus (Kahn et al. 2008; Wang et al. 2016). In contrast, the posterior portion of the hippocampus, including the tail, is mainly linked to frontal and parietal cortical regions (Poppenk et al. 2013).

Not only the hippocampus but also the amygdala is involved in auditory cognition, and is particularly susceptible to sounds with a valence, such as vocalizations, crying or music (Kraus and Canlon 2012). Furthermore, the amygdala is sensitive to auditory fear conditioning (Holland and Gallagher 1999; Rasia-Filho et al. 2000; Savage and Ramos 2009) and relevance detection (Sander et al. 2003; Zald 2003), and has been shown to be implicated in the regulation of attention (Gallagher and Holland 1994; Pessoa 2011), arousal (Davis and Whalen 2001; Murray and Wise 2010) as well as to modulate neuroplastic processes in the auditory cortex (Kraus and Canlon 2012). Importantly, acoustic stimuli characterized by a negative connotation have also been shown to induce the release of stress hormones via the hypothalamic–pituitary–adrenal axis (Kraus and Canlon 2012), and long-term exposure to glucocorticoids is known to promote synaptic loss, neural shrinkage and general gray matter atrophy (Nichols et al. 2001; Sapolsky et al. 1990).

Based on literature reviewed above, it seems obvious that the amygdala–hippocampal system deserves serious consideration for a more complete understanding of the neural circuits involved in the generation and maintenance of tinnitus beyond the auditory cortex (Kraus and Canlon 2012; Zhang et al. 2019). In line with this, the automatic FreeSurfer-based tripartition of the bilateral hippocampus only resulted in between-group volume differences in the left hemisphere, and this distinctive anatomical pattern was restricted to the anterior compartments consisting of head and body parts. In unison with the hippocampal results, amygdala volume differences between the two groups were also exclusively found in the left hemisphere. While it was beyond the scope of this study to determine the cause of distinct results in the two hemispheres, the common volumetric between-group differences we found in the anterior parts of the hippocampus and in the amygdala are possibly due to close connections between these two medial temporal lobe structures (Kahn et al. 2008; Wang et al. 2016). Hence, it is possible that anatomically predetermined connectivity patterns may have contributed to mutual volumetric changes in both brain structures, which resulted in a joint dysfunctional unit. It is also conceivable that tinnitus-related distress activated the hypothalamic–pituitary–adrenal axis and stimulated glucocorticoid release from the adrenal gland, which bound to the respective receptors in the limbic system, including the hippocampus and the amygdala (Roozendaal et al. 1999; Wang et al. 2014). However, although these two tinnitus-related brain regions are important targets for glucocorticoids and stress, high cortisol levels are commonly associated with decreased and not increased gray matter volumes in the hippocampus and the amygdala, especially due to a degradation of dendrites and synapses (Tata and Anderson 2010). Furthermore, the volumetric indices of the amygdala and of the head and body of the hippocampus were not predictive of tinnitus-related distress (THI), which makes it difficult to believe that the anatomical modifications were determined by glucocorticoids.

Although the increased amygdala and hippocampus volumes we revealed in the TIHL group are in line with a previous anatomical study of Profant and colleagues (Profant et al. 2020), the interpretation of these results is not straightforward. Nevertheless, there are several other factors that may have contributed to these findings. A first consideration is that the increased volumes of the amygdala and hippocampus were possibly induced by a tinnitus-related overstimulation of auditory cortical fields due to maladaptive reorganization of tonotopic maps (Kraus and Canlon 2012; Okamoto et al. 2010). Since auditory stimulation is essential for the development of amygdala and hippocampal functions (Billig et al. 2022; Zhang et al. 2022), it might have been the case that auditory overstimulation promoted an upregulation of receptors due to changes in neurotransmission, or even a spreading of blood vessels, all this resulting in increased structural volumes (Wasserthal et al. 2014; Zhang et al. 2022). Despite this, it is also possible that the common structural changes of the amygdala and the hippocampus were related to the sustainability of the tinnitus symptoms which were possibly mediated by the reciprocal dependence between the two brain structures (Billig et al. 2022). In this light, it might be that amygdala and hippocampus merged to a dysfunctional circuitry that triggered the maintenance of tinnitus through an amygdala-dependent orientation of attention (Gallagher and Holland 1994; Rasia-Filho et al. 2000), arousal (Davis and Whalen 2001; Murray and Wise 2010) or emotional appraisal toward the phantom sensations which benefited the long-term memory storage of tinnitus-related auditory information in the hippocampus (De Ridder et al. 2006; Naber et al. 2000).

Finally, it is noteworthy to mention that the discrepancy that exists in the literature regarding increased or decreased gray matter parameters and tinnitus results in general might be related to the high inter-individual variability of symptom manifestations (Cederroth et al. 2019) as well as to personal experiences in dealing with the syndrome. Therefore, future studies should mandatorily take into account the individual facets of tinnitus by combining functional and structural imaging protocols with comprehensive psychometric measurements. In this context, a major breakthrough in the field of pure-tone tinnitus research could be achieved, for example, using a multistep approach consisting of determining the individual tinnitus frequencies, of identifying the individual functional matrices responsive to pure-tone stimulation in the same frequency range, and of extracting and comparing gray matter parameters of the brain regions showing the highest degree of functional overlap. We are confident that such a procedure will open new avenues that are expected to enrich the understanding of tinnitus as a syndrome.

## Conclusions

Previous anatomical studies mostly examined tinnitus in isolation without considering auditory phantom sensations as part of a syndrome in comorbidity with pure-tone hearing loss. Furthermore, none of the previous studies took into account the multidimensional facets of hearing, and matched the groups for pure-tone hearing loss as well as for supra-threshold hearing estimates and SiN performance. Using a ROI approach, we identified the PT, the STS and the TPJ as fundamental hallmarks of the tinnitus syndrome matrix, which operates in interaction with other auxiliary brain regions. This specific pattern of outcomes leads us to believe that the PT plays a critical role in evoking the sensation of pure-tone ghosting, the TPJ in reinforcing the arousal drive for tinnitus-related features, and the STS in binding together all the multifaceted dimensions. Furthermore, the amygdala–hippocampus complex and the PFC might contribute to the regulation of valence and arousal levels. Finally, it is noteworthy to emphasize that due to the close spatial correspondence between-group-level results and brain–behavior relationships in the middle-anterior portion of the left STS, this multimodal territory meets the requirements of being a core area of the tinnitus syndrome matrix.

## Data Availability

We reported all measures and conditions without data exclusions. All codes and data are available from MM and RS. Due to ethical considerations, the MRI data cannot be made openly available. However, upon reasonable request, the MRI and behavioral data will be shared without any restrictions.
